# Retrospective Case Series of Ocular Lyme Disease, 1988–2025

**DOI:** 10.3201/eid3201.250769

**Published:** 2026-01

**Authors:** Jenna Bellafiore, Abdallah Mahrous, Vaishnavi Gurumurthy, Eugene Capitle, Steven E. Schutzer

**Affiliations:** Rutgers New Jersey Medical School, Newark, New Jersey, USA (J. Bellafiore, V. Gurumurthy, E. Capitle, S.E. Schutzer); Weill Cornell Medical College, New York, New York, USA (A. Mahrous)

**Keywords:** Lyme disease, Borrelia burgdorferi, bacteria, bacterial infections, uveitis, optic neuritis, cranial nerve palsy, ocular infection, vector-borne infections

## Abstract

Reports of ocular manifestations of Lyme disease (LD) are uncommon, and signs and symptoms may be overlooked by physicians. We conducted a retrospective case series of ocular LD reported during 1988–2025. Among 27 published reports in PubMed, we noted that, in 38 cases, the most common ocular manifestation was uveitis, representing 45% of cases, followed by optic neuritis and cranial nerve palsies (including trochlear and abducens). Not all cases met Centers for Disease Control and Prevention surveillance guidelines for LD, given that some case reports were published before the current guidelines. Cases that provided microbiologic proof were 2 anterior uveitis cases, 1 case of anterior uveitis with abducens’s nerve palsy, 1 case of intermediate uveitis, and 1 case of intranuclear ophthalmoplegia. Ocular LD can have a broad variety of manifestations; therefore, physicians should be aware of those manifestations and obtain microbiologic proof for a more definitive diagnosis and epidemiologic value when possible.

Lyme disease (LD), caused by *Borrelia burgdorferi*, is the leading vectorborne disease in the United States (*1*,*2*), transmitted by *Ixodes* ticks (*3*). LD has been described conceptually in stages, although manifestations of what was described as later stages can occur at the initial stage of infection. The initial stage often manifests with a characteristic skin rash, erythema migrans, described as a centrifugally expanding erythematous annular skin lesion with a clear center, or the bull’s-eye lesion, at the site of the tick bite (*4*). However, the classic form of erythema migrans does not occur in many cases, even those with microbiologic proof of the infection (*5**,**6*). The second stage of disease, occurring weeks to months later, and third stage of disease, occurring months later, are known to have a wide variety of manifestations, including neurologic, cardiac, and musculoskeletal signs and symptoms (*1*,*4*). Arthralgias are common in early LD, whereas arthritis, when it occurs, appears in later stages (*7*). In addition, various ocular etiologies have been observed during the second and third stages of LD. 

Although reports of ocular LD are rare, it can manifest in various ways, including, but not limited to, uveitis; optic neuritis; cranial nerve III, IV, and VII palsies; papilledema; and retinal vasculitis. We reviewed previously described case reports of ocular LD, summarizing the clinical manifestations to further clarify the possible manifestations of ocular LD to help guide physicians regarding when to consider this diagnosis. A caveat is that many of the early published cases did not necessarily follow the Centers for Disease Control and Prevention (CDC) case definitions at the time they were reported. Those definitions are for surveillance and not meant as clinical criteria for individual diagnosis and treatment (*8*,*9*).

## Methods

This retrospective case series aimed to evaluate ocular manifestations of LD by reviewing cases identified in the PubMed database that were published during 1988–2025. We included published articles in PubMed up until March 15, 2025, that described clinical manifestations of various forms of ocular LD. We conducted the literature search in PubMed by using the search terms “ocular Lyme” and “ocular and variations, with *B. burgdorferi*.” Inclusion criteria included articles that discussed >1 case report of ocular manifestations of LD. Exclusion criteria included inability to obtain full text, text in language other than English, and studies that did not discuss clinical manifestations of a specific case. We noted a paucity of articles that met CDC criteria at the time the cases were reported. Despite those limitations, we were able to illustrate the specific variety of ocular conditions by screening 176 articles; among the reviewed full texts, 29 were eligible for sufficient analysis. We excluded 2 texts because no English versions of the texts were available. We reviewed 27 publications (Table; Appendix Table). In order of decreasing assurance that the patient had LD were microbiologic evidence (e.g., DNA) or culture-positive test results for *B. burgdorferi*, meeting CDC criteria for LD with serologic conversion, CDC criteria otherwise being met, and CDC criteria not being met.

**Table:** Published case reports of ocular Lyme disease that indicate microbiologic proof of Lyme disease, 1988–2025*

Article authors	Year†	Evidence	Age/sex	Ocular manifestations, diagnosis, and additional symptoms	Treatment and resolution time in article
Dietrich et al. (*10*)	2008	Corneal specimen: spirochete-like bodies and fragments detected by light and electron-microscopic examination. PCR: positive for *Borrelia burgdorferi* sensu lato DNA. IFA: borderline. Western blot: weak reaction.	67/M	History of recurrent iridocyclitis and arthritis (unknown etiology) treated with methotrexate and steroids; developed progressive asymmetric keratopathy	Penetrating keratoplasty 2 times. IV ceftriaxone for 2 wks, and systemic immunosuppression (prednisone and methotrexate) continued. Tetracycline eyedrops and steroid eyedrops continued for >2 y without recurrence.
Hilton et al. (*11*)	1996	Vitreous fluid: positive PCR test result for 232-bp segment specific for *B. burgdorferi*; ELISA-negative (repeat test 4 mo later positive); Western blot negative, with faint reactivity to 4 IgG bands (repeat test 4 mo later positive).	26/F	Diagnosed with pars planitis	Doxycycline 100 mg 2×/d with improvement but recurrence. Treated with IV ceftriaxone 2 g/d for 10 d, followed by 2 mo oral macrolides. Visual deterioration requiring vitrectomy.
Kauffmann and Wormser (*12*)	1990	IFA: positive IgM and IgG. Vitreous debris examination showed occasional intact spirochetes compatible with Lyme disease. FTA-ABS and VRDL negative for *Treponema pallidum*.	45/F	Painful red eye with decreased vision and periorbital edema; diagnosed with iritis and posterior synechiae; additional symptoms: headache, lightheadedness, fevers, nausea, vomiting, EM-like rash	Prior treatment with steroids with development of sudden rise in ocular pressure with proptosis, conjunctival purulent discharge, and rapid-onset dense cataract. Started on nafcillin and gentamicin for possible orbital cellulitis. Without improvement, had vitrectomy 2 times.
Sauer et al. (*13*)	2009	ELISA: positive. Western blot: positive; aqueous humor: *Borrelia* spp. DNA noted.	39/F	Acute diplopia, pain and redness; diagnosed with abducens nerve palsy and anterior uveitis; additional symptoms: EM and arthralgia	Ceftriaxone 2 g/d for 2 wks and topical steroids with recovery.
Hardon et al. (*14*)	2002	ELISA-positive for IgG. CSF PCR positive for *Borrelia* spp. CSF antibody: negative.	31/M	Reduced eye movements; diagnosed with bilateral internuclear ophthalmoplegia	IV ceftriaxone 2 g/d for 3 wks with resolution.

*As of March 15, 2025. Year listed is the year of publication unless the year of the case is otherwise specified in the cited article. Not all cases were based on current Centers for Disease Control and Prevention case definition. CSF, cerebrospinal fluid; EM, erythema migrans; FTA-ABS, fluorescent treponemal antibody absorption test; IFA, indirect immunofluorescence assay; IV, intravenous; VDRL, Venereal Disease Research Laboratory test.

## Results

The reviewed literature (Table; Appendix Table) highlights diverse ocular manifestations of LD and the basis for their diagnosis and method for treatment. Of the 38 cases we analyzed, 5 cases had microbiologic proof of LD (*10*–*14*) (Table). Cases that had microbiologic proof were 2 cases of anterior uveitis, 1 case of intermediate uveitis, 1 case of abducens nerve palsy with anterior uveitis, and 1 case of intranuclear ophthalmoplegia.

### Ocular LD Cases with Microbiologic Proof

One case occurred in a 67-year-old man who had bilateral, progressive, asymmetric crystalline keratopathy. The patient had been taking methotrexate and systemic steroids for several years for recurrent iridocyclitis and arthritis of unknown cause. The patient had a penetrating keratoplasty of the right eye because of decreased visual acuity and had recurrence of the crystalline keratopathy 6 months later. Another 6 months later, he experienced acute vision loss accompanied by massive crystalline deposits. Another keratoplasty was performed, and a corneal specimen had spirochete-like bodies detected by light and electron microscopic examination and broadrange (16S rDNA) PCR tests that were positive for *B. burgdorferi* sensu lato DNA. The patient was then treated with intravenous ceftriaxone for 2 weeks and was continued on his immunosuppression agents; he also received tetracycline eyedrops and steroid eyedrops, which were continued for over 2 years. The corneal findings remained unchanged.

Another case occurred in a 26-year-old woman with unilateral intermediate uveitis, specifically pars planitis. Her vitreous fluid tested PCR-positive for *B. burgdorferi*. She was treated with oral doxycycline (100 mg 2×/d) and 2 months later had onset of keratitis and inflammation in the other eye. She was then started on intravenous ceftriaxone and experienced substantial improvement in her symptoms. However, 10 days later, after she had onset of severe thrombocytopenia, ceftriaxone was discontinued, and she was started on oral nitrofurantoin therapy for 2 months. She had continual visual deterioration, and a vitrectomy was performed. The vitreous fluid was found to be PCR-positive for a 232-bp segment specific for *B. burgdorferi*.

Another case with microbiologic proof occurred in a 45-year-old women who initially had systemic symptoms of fever, chills, headache, light headedness, and an erythema migrans–like rash. Approximately 30 days later, she had iritis with posterior synechiae. For the iritis, she received a subconjunctival injection of triamcinolone (40 mg) but then had onset of hypopyon with vitritis. She was then started on oral prednisone therapy (up to 100 mg/d), but the inflammation worsened, and she had onset of severe panophthalmitis. She then had a sudden rise in intraocular pressure with proptosis and a conjunctival purulent discharge and was started on nafcillin and gentamicin for orbital cellulitis. One week later, she had 1 lensectomy and 2 vitrectomy procedures; the specimen obtained during the second vitrectomy was stained by using the Deiterle method and showed occasional intact spirochetes on microscopic examination. A fluorescent treponemal antibody absorption test and a Venereal Disease Research Laboratory test showed that the specimen was negative for *Treponema pallidum* (the bacteria that causes syphilis).

Another case with microbiologic proof occurred in a 39-year-old woman who had a history of recent tick bite (within 3 months); a history of erythema migrans rash, arthralgia, and acute diplopia; and pain and redness in 1 eye. She was found to have abducens nerve palsy with anterior uveitis. Workup showed *Borrelia* spp. DNA in the aqueous humor specimen. She was treated with topical steroids and ceftriaxone for 2 weeks, during which time the patient recovered.

Another case in this series of cases with microbiologic proof of LD occurred in a 31-year-old man who had bilateral intranuclear ophthalmoplegia. A lumbar puncture was performed, and the result of a PCR test of the cerebrospinal fluid (CSF) was positive for *Borrelia* spp*.* The patient was treated with intravenous ceftriaxone for 3 weeks, and symptoms resolved. CSF from a repeat lumbar puncture after 3 months tested negative for *Borrelia* spp. by PCR.

### Possible or Probable Ocular LD Cases

Although some case reports showed microbiologic proof of LD through testing of CSF or specimens from the eye, many of the cases we found in the literature were diagnosed on the basis of laboratory results or erythema migrans in the setting of a recent tick bite. We assessed all the reported cases of possible or probable ocular LD (Appendix Table). Some of the cases followed the 2-tier or modified 2-tier testing method for LD; however, not all cases followed this method for diagnosis. Of all the case reports we reviewed (Table; Appendix Table), the most common ocular manifestation noted was uveitis (reported in 17 patients). Another common manifestation was cranial nerve palsies, which affect ocular movement (7 patients had abducens nerve palsy, and 2 patients has trochlear nerve palsy). Another 8 patients were found to have optic neuritis, of whom 4 had papillitis. Two patients had retinal vasculitis, and 2 patients had optic disc edema. One patient had scleritis. Additional symptoms noted in only 1 case report each included 1 case of ocular muscle myositis, 1 case of papilledema, 1 case of optic disc edema, 1 case of interstitial keratitis, 1 case of ocular flutter, 1 case of opsoclonus, and 1 case of internuclear ophthalmoplegia. Systemic symptoms such as fatigue, arthralgia, and influenza-like illness were frequently observed, indicating the multisystemic nature of LD.

## Discussion

Reports of ocular involvement in LD are relatively rare; such cases are sometimes linked to early infiltration into the eye by *B. burgdorferi* bacteria or *B. burgdorferi* remaining dormant in the eye and then manifesting with symptoms later (*4*). The reported cases we describe do not include a reaction of the eye from a tick bite occurring on or around the eye. Ocular LD demonstrates a broad spectrum of manifestations, most commonly various forms of uveitis (anterior, intermediate, posterior, and panuveitis) but also cranial nerve palsies, optic neuritis, retinal vasculitis, scleritis, and other rare ocular findings, often accompanied by systemic symptoms such as fatigue, arthralgia, and influenza-like illness, which underscore LD’s multisystemic nature. Of the 38 cases we reviewed, only 5 had definitive microbiologic confirmation through PCR or culture, whereas the remainder were classified as probable or possible on the basis of clinical features and serologic testing, which varied in consistency, reflecting historical diagnostic challenges.

First reported in the 1980s, shortly after *B. burgdorferi* was identified as the causative agent, ocular LD was initially described in case reports of conjunctivitis, uveitis, optic neuritis, and cranial nerve palsies. Certain cases of uveitis may be confused with conjunctivitis (pink eye) (Figure). Although diagnostic methods advanced in the 1990s and 2000s with improved serologic assays and PCR testing, microbiologic proof for ocular LD cases has remained rare. Our findings are consistent with previous literature, which has also documented the diversity of manifestations, the predominance of uveitis, the rarity of microbiologic confirmation, and variability in adherence to diagnostic guidelines, particularly in older reports. Similar studies include systematic reviews by Lu and Zand in 2022 (*1*), which analyzed LD-associated optic neuritis; Klaeger and Herbort in 2010 (*3*), which focused on retinal vascular changes; Johnson et al. in 2018 (*7*), which studied broader LD manifestations, including ocular findings; and multiple case series from the 1980s through 2000s, such as Fatterpekar et al. in 2002 (*4*) describing orbital LD (*1*,*3*,*4*,*7*).

**Figure:**
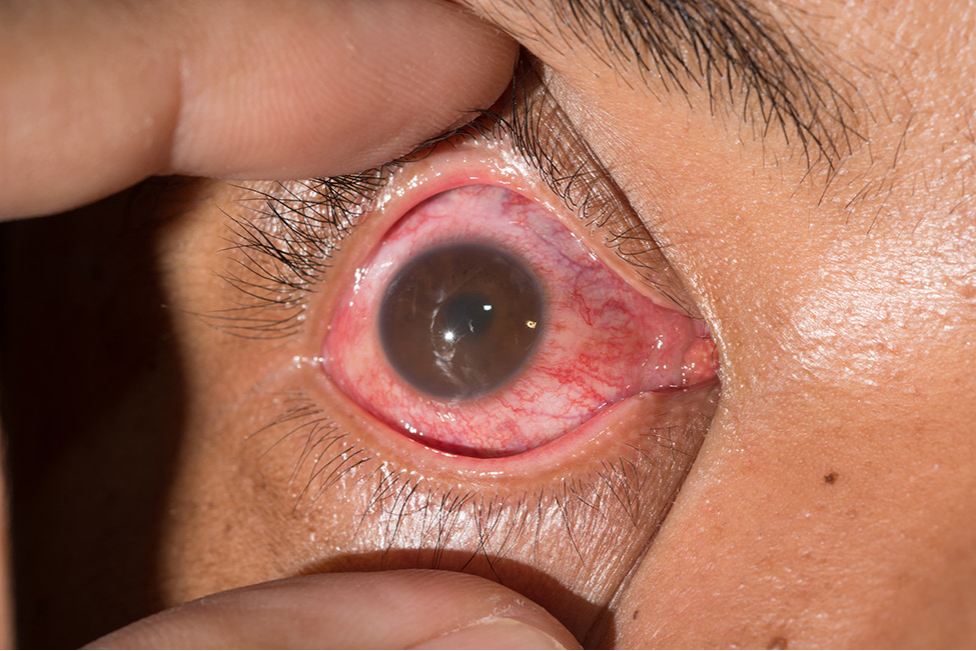
Anterior uveitis in a patient from a series of cases of ocular Lyme disease. Uveitis may be distinguished from the more common conjunctivitis (pink eye) by any number of features that warrant an ophthalmology examination. Uveitis is more likely to have eye pain, light sensitivity, smaller or irregular-shaped pupil, blurry vision, and floaters.

The heterogeneity of ocular manifestations found in patients with microbiologic proof of LD highlights the importance of testing for *B. burgdorferi* in patients with otherwise unexplained ocular manifestations. In addition, very few publications provided direct microbiologic proof of LD. Ideally, to recognize the possible ocular manifestations of LD, the presence of microbiologic proof of *B. burgdorferi* in some part of the body would be necessary.

Limitations of this study include the lack of microbiologic proof in most cases, heterogeneity in diagnostic criteria, small sample size, retrospective design with reliance on published case reports susceptible to reporting bias, incomplete or inconsistent clinical data, and temporal variability, given that many cases were published decades ago before modern diagnostic tools and treatment protocols. Those factors limit generalizability of case report findings to current clinical practice.

This comprehensive but limited analysis of published cases highlights clinical symptoms of possible or probable cases of LD. The successful outcomes in most cases, despite some requiring prolonged or repeated treatment, underscore the necessity of a multidisciplinary approach. Going forward, more comprehensive descriptions of ocular involvement should be published. This retrospective case series highlights the importance of further studies that can provide direct microbiologic proof for diagnosis of LD and guide the treatment of ocular manifestations in LD. Those measures will help us determine if ocular LD is an emerging condition.

In summary, LD can have, albeit rarely, a wide variety of ocular manifestations, most commonly uveitis, cranial nerve palsies, and optic neuritis. When evaluating a patient who lives or travels in an area of high LD prevalence, keeping LD in the differential diagnosis is important. A patient may seek primary care and clinicians in variety of subspecialties such as rheumatology, ophthalmology, infectious diseases, and neurology because of the various clinical manifestations of the illness. Therefore, all physicians need to be aware of the possibility of LD and be knowledgeable of how to test for it (or be ready to refer the patient to a colleague with expertise in LD) and report such cases to public health officials (*6*).

Appendix Additional information about retrospective case series of ocular Lyme disease, 1988–2025.
