# Supplementary material for: Retrospective Case Series of Ocular Lyme Disease, 1988–2025
Source: Emerg Infect Dis. 2026 Jan;32(1):15–20. doi: 10.3201/eid3201.250769 (PMC12870142; doi:10.3201/eid3201.250769)
Supplement: Appendix — Additional information about retrospective case series of ocular Lyme disease, 1988–2025. [file 25-0769-Techapp-s1.pdf]

Article DOI: https://doi.org/10.3201/eid3201.250769

EID cannot ensure accessibility for supplementary materials supplied by authors. Readers who have difficulty accessing supplementary content should contact the authors for assistance.

Retrospective Case Series of Ocular Lyme Disease, 1988–2025

Appendix

Appendix Table. Case reports of published cases of ocular Lyme disease

Article	Year*	Evidence	Age	Sex	Ocular Presentation / Diagnosis	Additional Symptoms	Treatment /resolution time in article
Havuz (*1*)	2021	First-tier Antibody: IgM positive, IgG negative<br>Western Blot: positive	21	M	Sudden onset monocular vision loss<br>Unilateral chorioretinitis	Fatigue, malaise	Doxycycline 100mg BID for 21 d Prednisone 1mg/kg/day for 10 d with resolution of symptoms
Patterson-Fortin J (*2*)	2016	Western blot: Positive IgM<br>Repeat Western blot: Positive IgM and IgG	80	F	Subacute, central monocular vision loss<br>Bilateral optic neuritis left more than right		Oral steroids for 2 weeks before initial presentation in hospital followed by 3 d of 1g solumedrol for 3 d followed by outpatient taper of oral steroids for possible giant cell arteritis<br>Steroids discontinued when diagnosis of Lyme made, then started on IV Ceftriaxone 2g BID for 5 weeks without visual improvement but with improvement in optic disc edema
McVeigh (*3*)	2012	ELISA antibody testing: Positive<br>Western blot: Positive	48	M	Decreased visual acuity and painful ocular movements, photophobia, inferotemporal vision distortion<br>Diagnosed with papillitis	Headache, mild ataxia	IV methylprednisolone 1g daily for 3 d, followed by oral prednisolone 40 mg daily, tapered by 5 mg weekly.<br>Oral amoxicillin 500 mg BID for 2 mo, IV ceftriaxone 2 g BID and oral doxycycline for 28 d. Visual acuity improved, disc swelling resolved
Mcveigh (*3*)	2012	History of tick bite<br>Western Blot: positive	79	M	Blurry vision transforming into “gray film” lasting a few minutes<br>Diagnosed with papillitis	Headache, tiredness, dizziness	Intravenous ceftriaxone 2 g daily for two weeks with resolution
Mahne (*4*)	2015	CSF Antibody: Positive IgM and IgG	13	M	Red left eye with painful eye movements, blurry vision, photophobia and increased lacrimation<br>Diagnosed with unilateral panuveitis with optic disc edema		IV ceftriaxone 2 g daily for 14 d, systemic acetazolamide with resolution
Ferro Desideri (*5*)	2023	First-tier Antibody: Positive	85	F	Unilateral decreased vision	Flu-like symptoms	Initially treated for suspected toxoplasma-associated uveitis

Article	Year*	Evidence	Age	Sex	Ocular Presentation / Diagnosis	Additional Symptoms	Treatment /resolution time in article
		Western Blot: Positive			Diagnosed with unilateral panuveitis with marked vitritis and patchy yellow lesions and retinal vasculitis		with oral TMP/SMX 1g BID, and anti-hypertensive acetazolamide 250 mg bid<br>After diagnosis started on Doxycycline 100mg BID and oral prednisone 50mg for 2 d with steroid taper.<br>With resolution of symptoms.
Kilic (*6*)	2016	First-tier Antibody: IgM positive<br>Western blot: Positive	30	M	Intermediate uveitis with retinal vasculitis, snowball opacities and multifocal dots (white dot syndrome)	Arthralgia, atypical rash	IV Ceftriaxone for 10 d followed by a 4-week course of amoxicillin/clavulanate and oral corticosteroid therapy with resolution.
Krist (*7*)	2002	ELISA: positive IgM<br>Lymphocyte Transformation Test: Positive<br>History of EM in the last few years	39	M	1 mo of slowly progressing pain in the left eye; Diagnosed with posterior scleritis.		Started on methylprednisolone 160mg daily with resolution in symptoms.<br>Patient declined antibiotics as symptoms has resolved.
Kauffmann (*8*)	1982	IFA: Positive IgM and IgG<br>Vitreous debris examination showed occasional intact spirochetes compatible with Lyme.<br>Syphilis (FTA-ABS and VRDL) negative.	45	F	Painful red eye with decreased vision and periorbital edema; Diagnosed with iritis and posterior synechiae	Headache, lightheadedness, fevers, nausea, vomiting, erythema-migrans like rash	Prior treatment with steroids with development of sudden onset rise in ocular pressure with proptosis, conjunctival purulent discharge and rapid onset dense cataract.<br>Started on Nafcillin and gentamicin for possible orbital cellulitis.<br>Without improvement, had vitrectomy x 2
Issa (*9*)	2021	Western Blot: Positive IgG and IgM<br>Repeat Western Blot 3 mo later: Positive IgG and IgM	33	F	Central blurred vision unilateral; Diagnosed with chorioretinitis		Prednisone 50mg daily and IV Ceftriaxone 2g BID for 4 weeks with steroid taper. Three months later, had a recurrence and restarted prednisone 50mg and Doxycycline 100mg BID for 2 mo with prednisone taper.<br>After treatment, the left eye worsened and restarted on prednisone 50mg, received dexamethasone intravitreal implant 0.7mg and then increased prednisone to 60mg, and started on Azithromycin 500mg daily for 3 mo and Doxycycline 100mg BID for 5 mo. Additionally started on Humira for steroid sparing agent. Prednisone tapered.<br>She finished her azithromycin, Doxycycline and prednisone courses and kept on adalimumab with improvement in symptoms and without recurrence
Dietrich (*10*)	2008	Corneal specimen: spirochete-like bodies and fragments were detected by light and electromicroscopy.	67	M	History of recurrent iridocyclitis and arthritis (unknown etiology) on methotrexate and steroids developed progressive		Penetrating keratoplasty x2.<br>IV Ceftriaxone x 2 weeks and systemic immunosuppression (prednisone and methotrexate) continued. Tetracycline eye drops and steroid eye drops continued for >2 y without recurrence.

Article	Year*	Evidence	Age	Sex	Ocular Presentation / Diagnosis	Additional Symptoms	Treatment /resolution time in article
		PCR: Positive for Borrelia burgdorferi sensu lato DNA IFA: Borderline Western Blot: Weak Reaction			asymmetric keratopathy.		
Lu (*11*)	2022	First-tier testing: Positive IgM CSF Antibody: IgM positive Western Blot: positive	48	F	History of multiple sclerosis presented with photophobia, eye pressure, blurry vision and painful eye movements; Diagnosed with bilateral optic neuritis	Fever, sore throat	IV methylprednisolone 1g/day x 3 d and then Ceftriaxone 2g / day for 25 d with resolution.
Burakgazi (*12*)	2016	CSF Antibody: Positive IgG Western Blot: Positive IgM and IgG	59	F	Decreased unilateral visual acuity and visual field defect; Diagnosed with unilateral optic neuritis	Rash, tiredness, arthralgias	Doxycycline 100mg BID for 6 weeks with resolution.
Miyashiro (*13*)	1999	ELISA: Positive	57	M	Foreign body sensation in the right eye; Diagnosed with unilateral interstitial keratitis		Tetracycline 250mg QID for 21 d with resolution
Moynagh (*14*)	2011	"Appropriate culturing blood tests" And possibly erythema migrans rash	22	M	Intermittent diplopia; Diagnosed with left partial sixth nerve plasy	Rash possibly erythema migrans	Symptoms self resolved after 6 weeks, treated after with 4 weeks IV Ceftriaxone
Sauer (*15*)	2009	ELISA: Positive Western Blot: Positive	4	M	Acute diplopia; Diagnosed with abducens nerve palsy	Erythema Migrans and arthritis	Ceftriaxone 2g / day for 3 weeks with recovery
Sauer (*15*)		ELISA: Positive Western Blot: Positive	46	F	Acute diplopia; Diagnosed with abducens nerve palsy	Erythema migrans, vestibular neuritis and arthralgia	Doxycycline 200mg / day for 2 weeks With recovery
Sauer (*15*)		ELISA: Positive Western Blot: Positive	34	M	Acute diplopia; Diagnosed with abducens nerve palsy	Erythema migrans and arthritis	Doxycycline 200mg / day for 2 weeks With recovery
Sauer (*15*)		ELISA: Positive Western Blot: Positive	61	M	Acute diplopia; Diagnosed with abducens nerve palsy and facial nerve palsy	Erythema Migrans	Ceftriaxone 2g / day for 3 weeks and oral steroids (1mg/kg/day for 7 d) Required botox muscle injection into the medial rectus muscle
Sauer (*15*)		ELISA: Positive Western Blot: Positive Aqueous Humor: *Borrelia* DNA noted	39	F	Acute diplopia, pain and redness; Diagnosed with abducens nerve palsy and anterior uveitis	Erythema Migrans and arthralgia	Ceftriaxone 2g / day for 2 weeks with recovery
Winward (*16*)	1988	IFA - Positive IgG ELISA - Positive	71	F	Oblique diplopia with right facial weakness and hypesthesia	Rash, malaise, fatigue, low grade fever	Prednisone for 3 d followed by doxycycline 300mg / day with recovery

Article	Year*	Evidence	Age	Sex	Ocular Presentation / Diagnosis	Additional Symptoms	Treatment /resolution time in article
					Diagnosed with right trochlear and facial nerve palsy		
Winward (*16*)	1988	IFA - Negative ELISA - Positive Western Blot: Positive	25	M	Blurry vision; Diagnosed with iridocyclitis and vitritis	Arthritis, recurrent facial nerve palsy	Failed topical steroids, oral doxycycline and erythromycin. Treated with IV Ceftriaxone 2g/day and IV Penicillin G 12 MU/day for 1 mo followed by 1 g/day of tetracycline for 9 mo with resolution. Additionally, recurrence occurred 1 mo later of bilateral iridocyclitis and vitritis with posterior synechiae treated then with cefuroxime 500mg / day with improvement. Also received 3 short courses of prednisone
Winward (*16*)	1975	IFA - Positive IgG ELISA - positive	26	F	Blurry vision and floaters; Diagnosed with vitritis with snowbanked exudates over the inferior pars plana	Rash, facial nerve palsy, flu-like symptoms, arthralgias, headache, fatigue	7 short courses of prednisone not described the time frame, IV Ceftriaxone 2g/day for 10 d without steroids with improvement in symptoms. Then recurrence after several months and started on IV Ceftriaxone 2g/day for 1 mo with recovery after a few months
Winward (*16*)	1988	IFA - negative ELISA - positive	11	F	Recurrent episodes of red, painful eyes; Diagnosed with iridocyclitis and vitritis bilaterally		Erythromycin for 1 mo without improvement. IV Ceftriaxone 1g/day for 1 mo with resolution
Winward (*16*)		IFA - positive x2	28	M	Red eyes, photophobia, and progressive visual loss; Diagnosed with iridocyclitis and vitritis bilaterally		IV Ceftriaxone 2g/day with 12MU/day of aqueous penicillin G for 7 d followed by 2g/day oral tetracycline for 3 weeks with resolution
Winward (*16*)	1987	IFA - negative ELISA - negative Western Blot - positive	23	F	Blurry vision in the bilateral eyes; Diagnosed with bilateral central scotomas, vitritis and optic neuritis	Peripheral neuropathy	Prednisone 60mg/day for 4 mo without improvement; IV ceftriaxone 1g/day for 5 d with improvement; symptoms recurred 7 mo later and treated with IV Ceftriaxone 3g/day for 3 weeks followed by oral erythromycin for several months with improvement
Jager (*17*)	2022	CSF antibody: Positive with positive CSF-to-serum antibody index	71	M	Involuntary back-to-back saccades in the horizontal plane; Diagnosed with ocular flutter	Gait ataxia	IV Ceftriaxone 2/g day for 8 d followed by Doxy 200mg/day for 13 d with resolution
Douglas (*18*)	20221	ELISA - positive Western blot: Positive IgG	24	F	Acute binocular diplopia when looking down and to the left; Diagnosed with unilateral trochlear nerve palsy	Fever, chills, back pain	Oral doxycycline for 30 d with resolution
Hilton (*19*)	1996	Vitreous Fluid: Positive PCR for 232 base-pair segment specific for *B burgdorferi*	26	F	Diagnosed with pars planitis		Doxycycline 100mg BID with improvement but recurrence. Treated with IV ceftriaxone 2g/day for 10 d followed by 2 mo of oral macrolides. Visual deterioration requiring vitrectomy

Article	Year*	Evidence	Age	Sex	Ocular Presentation / Diagnosis	Additional Symptoms	Treatment /resolution time in article
		ELISA - negative, repeat 4 mo later positive Western Blot - Negative (faint reactivity to 4 IgG bands); repeat 4 mo later positive					
May (*20*)	2021	ELISA – positive Western blot - positive with 6 IgG bands and 3 IgM bands	5	F	Photophobia, diplopia, eye pain and internal deviation of the right eye; Diagnosed with papilledema	Rash	Doxycycline for 21 d with resolution
Amer (*21*)	2009	ELISA - Negative Western blot - negative History of tick bite 3 mo earlier	16	M	Reduced right eye visual acuity; Diagnosed with papillitis, exudative macular lesion and active vasculitis in the posterior pole of the right eye and vasculitis of the left eye peripheral retinal vessels		IV Ceftriaxone 2g/day for 1 week and IV methylprednisolone 1g/day x 3 d with taper with resolution
Amer (*21*)		ELISA - negative History of tick bite	38	F	Reduced vision in the left eye; Diagnosed with papillitis, macular hemorrhages and vascular sheathing and development of inflammatory choroidal neovascular membrane	Rash, arthralgia	Doxycycline 100mg/day for 1 week before onset of ocular symptoms; IV Ceftriaxone 2g/day for 4 weeks with further decline in vision and with retinal fibrosis development; patient declined further treatment
Dabiri (*22*)	2018	ELISA - negative Western blot - Initial Negative (1IgG and 1IgM band) Repeat Western Blot 3 mo later: Positive 3 IgM bands positive CSF antibodies: Negative	56	F	Diplopia and blurry vision in the left eye; Diagnosed with left lateral rectus palsy (left abducens palsy) and mild left optic neuritis	Rash, unilateral hemifacial pain/headach e	Methylprednisolone dose pack (4mg tabs) without improvement. Doxycycline 100mg BID and 2 d later developed rash and started on Valacyclovir with improvement in rash after 2 d. Restarted later on Doxycycline 100mg BID for 12 weeks with improvement in symptoms
Klaegar (*23*)	2010	ELISA - Positive Western blot - positive IgG	52	F	Recurrent iritis unilateral	Migrating skin rash, headache	Doxycycline 100mg BID for 3 weeks with development of cotton wool spots and recurrence of anterior uveitis then treated with IV Ceftriaxone 2g /day x 3 weeks with resolution
Gibaud (*24*)	2019	Western blot - Positive CSF Antibody: positive	9	F	Anarchic, involuntary and multidirectional eye movements in both eyes with left upper and incomplete lower facial nerve palsy; Diagnosed with opsoclonus and	Febrile headaches	Ceftriaxone 2g/day for 3 weeks with resolution

Article	Year*	Evidence	Age	Sex	Ocular Presentation / Diagnosis	Additional Symptoms	Treatment /resolution time in article
					left facial nerve palsy		
Breeveld (*25*)	1987	IFA - positive	29	M	Chronic visual decline and new blurry vision and conjunctival redness; Diagnosed with chronic intermediate uveitis and associated pars planitis	Radicular pain, skin rash	IV Ceftriaxone 4g/day for 2 weeks with resolution
Fatterpekar (*26*)	2002	History of skin rash and living in endemic area	46	F	Double vision Diagnosed with ocular muscle myositis	Headache, rash, fever	Doxycycline 100mg BID for 3 weeks with resolution
Hardon (*27*)	2001	ELISA - Positive for IgG CSF PCR positive for *Borrelia burgdorferi* CSF Antibody: Negative	31	M	Reduced eye movements Diagnosed with bilateral internuclear ophthalmoplegia		Ceftriaxone IV 2g / day for 3 weeks with resolution

Abbreviations: ELISA = Enzyme-linked Immunosorbent Assay, IFA = Indirect Immunofluorescence assay; PCR = polymerase chain reaction, CSF = cerebral spinal fluid, BID = twice a day, QID = four times a day, TMP/SMX = trimethoprim-sulfamethoxazole, IV = intravenous.
*Year is the year of publication unless the year of the case is otherwise specified in the paper. Not all cases employed the current CDC Case Definition.

References

1. Havuz E, Güdül Havuz S. Lyme disease atypically presenting with a singular symptom: Unilateral chorioretinitis. Eur J Ophthalmol. 2021;31:NP151–6. PubMed https://doi.org/10.1177/1120672120962055

2. Patterson-Fortin J, Kohli A, Suarez MJ, Miller PE. Ocular Lyme borreliosis as a rare presentation of unilateral vision loss. BMJ Case Rep. 2016;2016:bcr2016215307. PubMed https://doi.org/10.1136/bcr-2016-215307

3. McVeigh K, Vakros G. Case report: papillitis as the sole ocular sign in Lyme disease. Clin Ophthalmol. 2012;6:1093–7. PubMed

4. Mahne J, Kranjc BS, Strle F, Ružić-Sabljić E, Arnež M. Panuveitis caused by *Borrelia burgdorferi* sensu lato infection. Pediatr Infect Dis J. 2015;34:102–4. PubMed https://doi.org/10.1097/INF.0000000000000473

5. Ferro Desideri L, Rosa R, Forte P, Manocchio R, Vagge A, Traverso CE, et al. Multimodal imaging for the management of Lyme-associated uveitis: A case report from an Italian tertiary center. Eur J Ophthalmol. 2023;33:NP58–64. PubMed https://doi.org/10.1177/11206721231154172

6. Kılıç Müftüoğlu İ, Aydın Akova Y, Gür Güngör S. A case of Lyme disease accompanied by uveitis and white dot syndrome. Turk J Ophthalmol. 2016;46:241–3. PubMed

7. Krist D, Wenkel H. Posterior scleritis associated with *Borrelia burgdorferi* (Lyme disease) infection. Ophthalmology. 2002;109:143–5. PubMed https://doi.org/10.1016/S0161-6420(01)00868-5

8. Kauffmann DJ, Wormser GP. Ocular Lyme disease: case report and review of the literature. Br J Ophthalmol. 1990;74:325–7. PubMed https://doi.org/10.1136/bjo.74.6.325

9. Issa R, DeSouza SAM. Recurrent bilateral chorioretinitis with positive Lyme serology: a case report. J Med Case Rep. 2021;15:253. PubMed https://doi.org/10.1186/s13256-021-02804-7

10. Dietrich T, Geissdörfer W, Schlötzer-Schrehardt U, Holbach L, Schoerner C, Seitz B. *Borrelia*-associated crystalline keratopathy with intracorneal detection of *Borrelia garinii* by electron microscopy and polymerase chain reaction. Cornea. 2008;27:498–500. PubMed https://doi.org/10.1097/ICO.0b013e318162a8f5

11. Lu Y, Zand R. Characteristics of Lyme optic neuritis: a case report of Lyme associated bilateral optic neuritis and systematic review of the literature. BMC Neurol. 2022;22:113. PubMed https://doi.org/10.1186/s12883-022-02627-z

12. Burakgazi AZ, Henderson CS. Unusual presentation of unilateral isolated probable Lyme optic neuritis. Case Rep Neurol Med. 2016;2016:7471842. PubMed https://doi.org/10.1155/2016/7471842

13. Miyashiro MJ, Yee RW, Patel G, Ruiz RS. Lyme disease associated with unilateral interstitial keratitis. Cornea. 1999;18:115–6. PubMed https://doi.org/10.1097/00003226-199901000-00017

14. Moynagh K-A, McNamara R. Ocular manifestations as a result of Lyme disease: a case report. Br Ir Orthopt J. 2011;8:66–8. https://doi.org/10.22599/bioj.45

15. Sauer A, Hansmann Y, Jaulhac B, Bourcier T, Speeg-Schatz C. Five cases of paralytic strabismus as a rare feature of lyme disease. Clin Infect Dis. 2009;48:756–9. PubMed https://doi.org/10.1086/597041

16. Winward KE, Smith JL, Culbertson WW, Paris-Hamelin A. Ocular Lyme borreliosis. Am J Ophthalmol. 1989;108:651–7. PubMed https://doi.org/10.1016/0002-9394(89)90856-8

17. Jäger F, Greisenegger S, Schwarz FK, Wiest G. Ocular flutter is a rare and treatment-responsive symptom in Lyme neuroborreliosis: a case report. J Neurol. 2022;269:1026–7. PubMed https://doi.org/10.1007/s00415-021-10769-2

18. Douglas VP, Douglas KAA, Reinshagen KL, Chwalisz BK. Case 292: Lyme Neuroborreliosis. Radiology. 2021;300:484–8. PubMed https://doi.org/10.1148/radiol.2021201715

19. Hilton E, Smith C, Sood S. Ocular Lyme borreliosis diagnosed by polymerase chain reaction on vitreous fluid. Ann Intern Med. 1996;125:424–5. PubMed https://doi.org/10.7326/0003-4819-125-5-199609010-00028

20. May K, Upadhyayula S. Papilledema as the sole manifestation of neuroborreliosis. Case Rep Infect Dis. 2021;2021:5565900. PubMed https://doi.org/10.1155/2021/5565900

21. Amer R, Brannan S, Forrester JV. Inflammatory choroidal neovascular membrane in presumed ocular Lyme borreliosis. Acta Ophthalmol. 2009;87:346–8. PubMed https://doi.org/10.1111/j.1755-3768.2007.01160.x

22. Dabiri I, Burakgazi AZ. Simultaneous involvement of optic and abducens nerves by Lyme disease: Case report with review of the literature. Neurol Int. 2019;11:8241. PubMed https://doi.org/10.4081/ni.2019.8241

23. Klaeger AJ, Herbort CP. Cotton wool spots as possible indicators of retinal vascular pathology in ocular lyme borreliosis. Int Ophthalmol. 2010;30:599–602. PubMed https://doi.org/10.1007/s10792-008-9268-5

24. Gibaud M, Pauvert O, Gueden S, Durigneux J, Van Bogaert P. Opsoclonus in a child with neuroborreliosis: Case report and review of the literature. Arch Pediatr. 2019;26:118–9. PubMed https://doi.org/10.1016/j.arcped.2018.11.013

25. Breeveld J, Rothova A, Kuiper H. Intermediate uveitis and Lyme borreliosis. Br J Ophthalmol. 1992;76:181–2. PubMed https://doi.org/10.1136/bjo.76.3.181

26. Fatterpekar GM, Gottesman RI, Sacher M, Som PM. Orbital Lyme disease: MR imaging before and after treatment: case report. AJNR Am J Neuroradiol. 2002;23:657–9. PubMed

27. Hardon WJ, Bernsen HJ, van Nouhuys-Leenders J, Mulder B. Internuclear ophthalmoplegia as the first sign of neuroborreliosis. J Neurol. 2002;249:1119–20. PubMed https://doi.org/10.1007/s00415-002-0747-8
